# Altered MicroRNA Maturation in Ischemic Hearts: Implication of Hypoxia on XPO5 and DICER1 Dysregulation and RedoximiR State

**DOI:** 10.3390/antiox12071337

**Published:** 2023-06-24

**Authors:** Lorena Pérez-Carrillo, Isaac Giménez-Escamilla, María García-Manzanares, Juan Carlos Triviño, Sandra Feijóo-Bandín, Alana Aragón-Herrera, Francisca Lago, Luis Martínez-Dolz, Manuel Portolés, Estefanía Tarazón, Esther Roselló-Lletí

**Affiliations:** 1 Clinical and Translational Research in Cardiology Unit, Health Research Institute Hospital La Fe (IIS La Fe), Avd. Fernando Abril Martorell 106, 46026 Valencia, Spain; lorena_perezc@iislafe.es (L.P.-C.); isaac_gimenez@iislafe.es (I.G.-E.); luismartinezdolz@gmail.com (L.M.-D.); portoles_man@gva.es (M.P.); 2 Center for Biomedical Research Network on Cardiovascular Diseases (CIBERCV), Avd. Monforte de Lemos 3-5, 28029 Madrid, Spain; garciamanzanares.maria@gmail.com (M.G.-M.); sandra.feijoo.bandin@sergas.es (S.F.-B.); alana.aragon.herrera@sergas.es (A.A.-H.); francisca.lago.paz@sergas.es (F.L.); 3 Medicine and Animal Surgery, Veterinary School, CEU Cardenal Herrera University, C/Lluís Vives, 1, 46115 Alfara del Patriarca, Spain; 4 Genomic Systems, Ronda de Guglielmo Marconi, 6, 46980 Paterna, Spain; jc.trivino@sistemasgenomicos.com; 5 Cellular and Molecular Cardiology Research Unit, Department of Cardiology and Institute of Biomedical Research, University Clinical Hospital, Tr.ª da Choupana, 15706 Santiago de Compostela, Spain; 6 Heart Failure and Transplantation Unit, Cardiology Department, University and Polytechnic La Fe Hospital, Avd. Fernando Abril Martorell 106, 46026 Valencia, Spain

**Keywords:** microRNA biogenesis, redoximiRs, ischemic cardiomyopathy, microRNA maturation

## Abstract

Ischemic cardiomyopathy (ICM) is associated with abnormal microRNA expression levels that involve an altered gene expression profile. However, little is known about the underlying causes of microRNA disruption in ICM and whether microRNA maturation is compromised. Therefore, we focused on microRNA maturation defects analysis and the implication of the microRNA biogenesis pathway and redox-sensitive microRNAs (redoximiRs). Transcriptomic changes were investigated via ncRNA-seq (ICM, *n* = 22; controls, *n* = 8) and mRNA-seq (ICM, *n* = 13; control, *n* = 10). The effect of hypoxia on the biogenesis of microRNAs was evaluated in the AC16 cell line. ICM patients showed a reduction in microRNA maturation compared to control (4.30 ± 0.94 au vs. 5.34 ± 1.07 au, *p* ˂ 0.05), accompanied by a deregulation of the microRNA biogenesis pathway: a decrease in pre-microRNA export (*XPO5*, FC = −1.38, *p* ˂ 0.05) and cytoplasmic processing (*DICER*, FC = −1.32, *p* ˂ 0.01). Both processes were regulated by hypoxia in AC16 cells (*XPO5*, FC = −1.65; *DICER1*, FC = −1.55; *p* ˂ 0.01; Exportin-5, FC = −1.81; Dicer, FC = −1.15; *p* ˂ 0.05). Patients displayed deregulation of several redoximiRs, highlighting miR-122-5p (FC = −2.41, *p* ˂ 0.001), which maintained a good correlation with the ejection fraction (r = 0.681, *p* ˂ 0.01). We evidenced a decrease in microRNA maturation mainly linked to a decrease in XPO5-mediated pre-microRNA export and DICER1-mediated processing, together with a general effect of hypoxia through deregulation of biogenesis pathway and the redoximiRs.

## 1. Introduction

Heart failure was defined as a global pandemic. It constitutes a significant economic and public health burden with high morbidity and mortality rates worldwide [1], mainly due to ischemic heart diseases, and is a leading global cause of death [2]. The pathological process of the heart is associated with an altered expression profile of molecules that are important for cardiac function [3,4,5,6]; however, the molecular mechanisms of gene regulation remain incompletely understood. Most human protein-coding genes are likely under microRNA control through microRNA-binding sites located in their untranslated and amino acid coding regions [7,8] and, therefore, microRNA are probably involved in nearly all developmental and pathological processes. Thus, it was described that alteration in the expression of microRNA contributes to a range of human pathologies, including heart failure [9,10]. However, little is known about the underlying causes of microRNA disruption and whether microRNA maturation is compromised. In cancer, it was proposed that the global dysregulation of microRNA mainly involves transcriptional control and/or impaired biogenesis [11].

microRNA biogenesis is tightly regulated at multiple levels and involves both nuclear and cytoplasmic processing [12]. Altered expression and mutations of members of the microRNA processing machinery can lead to changes ranging the transcription of microRNA, processing, export and maturation [11], leading to a marked reduction in the production of mature microRNAs [13]. Emerging evidence suggests that microRNA biogenesis can be affected by oxidative stress, an important pathophysiological pathway in the development and progression of heart failure, especially in ischemic heart disease [14]; in a complex interplay between microRNA and ROS in which microRNA also can regulate cellular redox status [15]. The number of identified redox-sensitive microRNA is increasing [16,17], but this relationship may be broader [18,19]. This complex network between microRNAs and oxidative stress acts by modulating cell homeostasis, but despite the fact that several genome-wide microRNA studies identified microRNA differentially expressed in heart failure [9,10], suggesting their possible involvement in the pathogenesis, the state of microRNA maturity in heart failure and how the processing machinery and redox state control it is unknown.

In this study, we analyzed the state of microRNA maturity and its biogenesis process in ischemic cardiomyopathy (ICM). We focused especially on redox-sensitive microRNAs alterations that occur in these patients. We further delved into how oxidative stress affects the molecular machinery that controls microRNA biogenesis. This study demonstrates that global microRNA maturation is dysregulated in human ICM and sheds new light on the causes and potential implications of redox-sensitive microRNAs (redoximiRs) dysregulation. Here, we provide evidence that both changes in the microRNA biogenesis pathway and redoximiRs, particularly regulation by hypoxia, could participate in microRNA maturation defects.

## 2. Materials and Methods

### 2.1. Tissue Samples and Patients

Myocardial tissue samples were obtained from near the apex of the left ventricle of patients diagnosed with ICM undergoing heart transplantation and control donors. Specifically, 30 samples were used for non-coding RNA sequencing (ncRNA-seq; ICM, *n* = 22; control, *n* = 8), and 23 samples were used for mRNA sequencing (mRNA-seq; ICM, *n* = 13; control, *n* = 10). Tissue samples were preserved in 0.9% NaCl at 4 °C for 4.4 ± 3 h after coronary circulation loss and were storage at −80 °C until use. A reduced time between sample receipt and stored produced higher-quality samples, as evidenced by the RNA integrity numbers of ≥9.

Patients were diagnosed based on clinical history and results from hemodynamic, electrocardiographic, Doppler echocardiography and coronary angiography studies. The ICM diagnoses were based on the following inclusion criteria: (i) there were prior documented episodes of acute myocardial infarction, (ii) the echocardiography showed normal contractility segments coexisting with other dyskinetic or akinetic segments and iii) the electrocardiography showed signs of ischemia or myocardial necrosis.

All patients were classified according to the New York Heart Association (NYHA) functional criteria and were receiving medical treatment according to the guidelines of the European Society of Cardiology [20]. They were previously diagnosed with significant comorbidities, including hypertension and type 2 diabetes. Table 1 summarizes their clinical characteristics.

All controls had normal left ventricular function (ejection fraction ≥ 50%) as determined by Doppler echocardiography, and they had no history of cardiac disease. The control samples were obtained from non-diseased hearts that could not be transplanted owing to surgical reasons or blood type incompatibility. The cause of death of these donors was a cerebrovascular event or a motor vehicle accident.

The investigation conforms to the principles outlined in the Declaration of Helsinki [21] and was approved by the Ethics Committee (Biomedical Investigation Ethics Committee of La Fe University Hospital of Valencia, Spain; protocol code 2016/0320, 15 November 2016). All participants signed their written informed consent to participate in the study.

### 2.2. RNA Extraction and Integrity

Heart samples were homogenized in a TissueLysser LT (Qiagen). RNA extractions were performed using a Quik-RNA^TM^ miniprep plus kit (Zymo Research) for non-coding RNA sequencing (ncRNA-seq; ICM, *n* = 22; control, *n* = 8) or a PureLink™ Kit (Ambion Life Technologies) for mRNA sequencing (mRNA-seq; ICM, *n* = 13; control, *n* = 10), according to the manufacturer’s instructions. RNA was quantified using a NanoDrop1000 spectrophotometer and at Qubit 3.0 fluorimeter (Thermo Fisher Scientific). The purity and integrity of RNA samples was measured using an Agilent 2100 Bioanalyzer with the RNA 6000 Nano LabChip kit (Agilent Technologies). All samples displayed a 260/280 absorbance ratio > 2.0 and RNA integrity numbers ≥ 9.

### 2.3. ncRNA-Seq Analysis

The cDNA libraries were obtained following Illumina’s recommendations. Briefly, 3′ and 5′adaptors were sequentially ligated to the RNA prior to reverse transcription and cDNA generation. The cDNA was enriched using PCR to create an indexed double-stranded cDNA library, and size selection was performed using a 6% polyacrylamide gel. The quality and quantity of the libraries were analyzed using a 4200 TapeStation D1000 High-Sensitivity assay. The cDNA libraries were pooled and the pools were sequenced using paired-end sequencing (100 × 2) in the Illumina HiSeq 2500 sequencer.

The quality control of the raw data was performed using the FastQC tool. For the adapter and quality filler of raw data, trim_galore was applied [http://www.bioinformatics.babraham.ac.uk/projects/trim_galore/] (accessed on 15 May 2020). Then, the insufficient quality reads (phred score < 20) were eliminated using the Picard Tools software [22]. RNAs predictions were estimated using HT Seq software (version 0.6.0) [23].

### 2.4. Mature/Immature microRNA Ratio

The raw data for each sample were mapped against the human mature and hairpin sequences contained in mirBase [24] using the bowtie algorithm [22]. The low-quality reads were filtered using a Q20 threshold. Finally, only the unique mapped reads were considered for the next analyses. The fraction of mature reads mapped versus hairpin precursors was calculated. The statistical difference of fraction between conditions was evaluated using Wilcoxon test [25].

### 2.5. mRNA-Seq Analysis

PolyA-RNA was isolated form 25 micrograms of total RNA using the MicroPoly(A) Purist kit (Ambion). Total Poly(A) RNA samples were used to generate whole transcriptome libraries for sequencing on the SOLiD 5500XL platform, following the manufacturer’s recommendation (Life Technologies). No RNA spike-in controls were used. Amplified cDNA quality was analyzed by the Bioanalyzer 2100 DNA 1000 kit (Agilent Technologies) and quantified using the Qubit 2.0 Fluorimeter (Invitrogen). The whole transcriptome libraries were used for making SOLiD templated beads following the SOLiD Templated Bead Preparation guide. Bead quality was estimated based on WFA (workflow analysis) parameters. The samples were sequenced using the 50,625 paired-end protocol, generating 75 nt + 35 nt (Paired-End) + 5 nt (Barcode) sequences. Quality data were measured using software SETS parameters (SOLiD Experimental Tracking System).

The initial whole transcriptome paired-end reads obtained from sequencing were mapped against the latest version of the human genome (version GRchr37/hg19) using the Life Technologies mapping algorithm (http://www.lifetechnologies.com/, accessed on 15 May 2020), version 1.3. We used the standard Bioscope parameters of version 1.3 in paired-ends and whole transcriptome analysis. For both reads, forwards and reverse, the seed was the first 25 nucleotides with a maximum of 2 mismatches allowed. The aligned records were reported in BAM/SAM format [26]. Bad quality reads (Phred score < 10) were eliminated using Picard Tools software, version 1.83 (http://broadinstitute.github.io/picard/, accessed on 15 May 2020).

The mRNA-seq data discussed in this publication were deposited in NCBI’s Gene Expression Omnibus [27] and are accessible through GEO Series accession number GSE55296 (http://www.ncbi.nlm.nih.gov/geo/query/acc.cgi?acc=GSE55296, accessed on 15 May 2020).

### 2.6. Identification of microRNA-mRNA and Protein–Protein Interactions

We investigated experimentally validated targets of redoximiRs, among the microRNA biogenesis-related genes differentially expressed by accessing data from the miRTarBase [28] and TaRbase [29] databases. These databases contain experimentally verified microRNA targets on coding and non-coding RNAs. Protein–protein interactions were also accessed through the STRING v11.5 software (available at https://string-db.org/, accessed on 2 February 2023). The parameters that were evaluated included experiments, databases and co-expression. Furthermore, we investigated the relationships in the expression of these molecules and we overlapped this information with the predicted target microRNAs and protein–protein interactions to construct a microRNA–target gene regulatory network.

### 2.7. Cell Culture

AC16 Human Cardiomyocyte Cell Line (SCC109, Merck, St. Louis, MO, USA) was cultured in DMEM/F12 medium (Gibco™, Thermo Fisher Scientific, Horsham, UK), supplemented with 2 mM L-Glutamine (Gibco™, Thermo Fisher Scientific), 12.5% fetal bovine serum (FBS, LINUS), 50 U/mL penicillin and 50 µg/mL streptomycin (Gibco™, Thermo Fisher Scientific, Horsham, UK), at 37 °C in a 5% CO_2_ incubator. The culture medium was replaced every two to three days until 90% cell confluence was reached and was used in the following experimental assays.

In total, 8 × 104 human cardiomyocytes per well were seeded in 6-well plates. The cells were cultured at 37 °C in hypoxic conditions (5% CO_2_ and 2% O_2_), simulating the myocardial hypoxia of ICM, or normoxic conditions as control. At 48 h, the cells were harvested and the expression of the target molecules was analyzed.

### 2.8. Determination of XPO5 and DICER1 Expression Levels

Total cell RNA was extracted using a Qiagen miRNeasy Mini kit (normoxia, *n* = 6; hypoxia, *n* = 6) following the manufacturer’s instructions (Qiagen) and was quantified using a NanoDrop 2000c spectrophotometer. For determination of *XPO5* and *DICER* expression levels, complementary DNA synthesis was carried out using M-MLV reverse transcriptase (Invitrogen) according to the manufacturer’s protocol. qPCR was performed using Taqman^®^ Gene Expression Assays (Applied Biosystems, Thermo Fisher Scientific, Horsham, UK) in a ViiA7 Real-Time PCR System (Applied Biosystems; Thermo Fisher Scientific, Horsham, UK) according to the manufacturer’s instructions. The following TaqMan probes were obtained from Thermo Fisher Scientific: *XPO5* (Hs00382453_m1), *DICER1* (Hs00229023_m1), and *GAPDH* (Hs99999905_m1). The average Ct of *GAPDH* was used to normalize *XPO5* and *DICER1* in each sample. The Fold Change 2^−∆∆Ct^ method was used to compare relative expression between samples of both conditions [30].

### 2.9. Determination of Exportin-5 and Endoribonuclease Dicer Protein Levels

Total cellular protein (normoxia, *n* = 6; hypoxia, *n* = 5) was extracted using total protein extraction buffer (2% SDS, 10 mM EDTA, 6 mM Tris–HCl, pH 7.4) supplemented with protease inhibitors (25 µg/mL aprotinin and 10 µg/mL leupeptin). Samples were sonicated and total protein was quantified by Peterson’s modification of the micro Lowry method (Sigma-Aldrich) with bovine serum albumin (BSA) as the standard.

The protein samples for detecting Exportin-5 and Endoribonuclease Dicer were subsequently separated by Bis-Tris 4–12% polyacrylamide gels under reducing conditions. After electrophoresis, proteins were transferred to a polyvinylidene difluoride membrane (PVDF) using the iBlot Dry Blotting System (Invitrogen Ltd., Waltham, MA, USA), blocked at 4 °C overnight with 1% BSA in Tris buffer solution containing 0.05% Tween 20 and were incubated for 2 hours with the primary antibody in the same buffer. The primary detection antibodies used were anti-Exportin-5 rabbit monoclonal antibody (1:1000; ab131281), anti-Dicer1 rabbit polyclonal antibody (1:1000; ab227518) and anti-GAPDH mouse monoclonal antibody (1:2000; ab8245) as a loading control, all obtained from Abcam.

The bands were visualized using an acid phosphatase-conjugated secondary antibody and nitro blue tetrazolium/5-bromo-4-chloro-3-indolyl phosphate (NBT/BCIP, Sigma-Aldrich, St. Louis, MO, USA) substrate system. Finally, the bands were digitalized using an image analyzer (DNR Bio-Imagining Systems, Jerusalem, Israel) and quantified with the GelQuant Pro (v. 12.2) program.

### 2.10. Statistical Methods

Data were expressed as the mean ± standard deviation for continuous variables and as percentage values for discrete variables. The Kolmogorov–Smirnov test was applied to analyze the data distribution. Clinical characteristics of patients were compared by using Student’s *t*-test for continuous variables and Fisher’s exact test for discrete variables. Differential ncRNA expression analysis between conditions was assessed using the DESeq2 method [31] (version 3.4). We considered those ncRNAs with a *p* value (P adj) corrected by FDR  ≤  0.05 as differently expressed to avoid identification of false positives across the differential expression data [32]. Gene predictions were estimated using the cufflinks method [33] and the expression levels were calculated using the HTSeq software, version 0.5.4p323. This method eliminated the multimapped reads, and only the unique reads were considered for gene expression estimation. The edgeR method, version 3.2.4, was applied for differential expression analysis between conditions [34]. This method relies on different normalizing processes based on in-depth global samples, CG composition and length of genes. In the differential expression process, this method relies on a Poisson model to estimate the variance of the RNA-seq data for differential expressions. Significant mean differences in protein levels were analyzed by using Student’s *t*-test for variables with a normal distribution and Mann–Whitney U test for variables with a non-normal distribution. Pearson’s correlation coefficient was calculated to analyze the association between variables with normal distribution and Spearman’s correlation coefficient for variables with non-normal distribution. *p* < 0.05 was considered statistically significant. All statistical analyses were performed using the SPSS software (version 20.0) for Windows (IBM SPSS Inc., Chicago, IL, USA).

## 3. Results

### 3.1. Patient Characteristics

The study populations for each assay (ncRNA-seq and mRNA-seq) were homogeneous based on the clinical characteristics of the patients. Table 1 shows the clinical characteristics of ICM patients of each assay. All of them were men; were classified as III–IV New York Heart Association functional classes; had several comorbidities, such as hypertension (33–40%) and diabetes (42–45%); and were mainly previous current smokers (81–92%). The control group was mainly men (62%) with a mean age of 50 ± 15 years. Comorbidities and other echocardiographic data were not available for the control group, in accordance with the Spanish Organic Law on Data Protection 15/1999.

### 3.2. Mature microRNA Expression Changes in ICM Patients: Implication of microRNA Biogenesis

**Figure 1:**
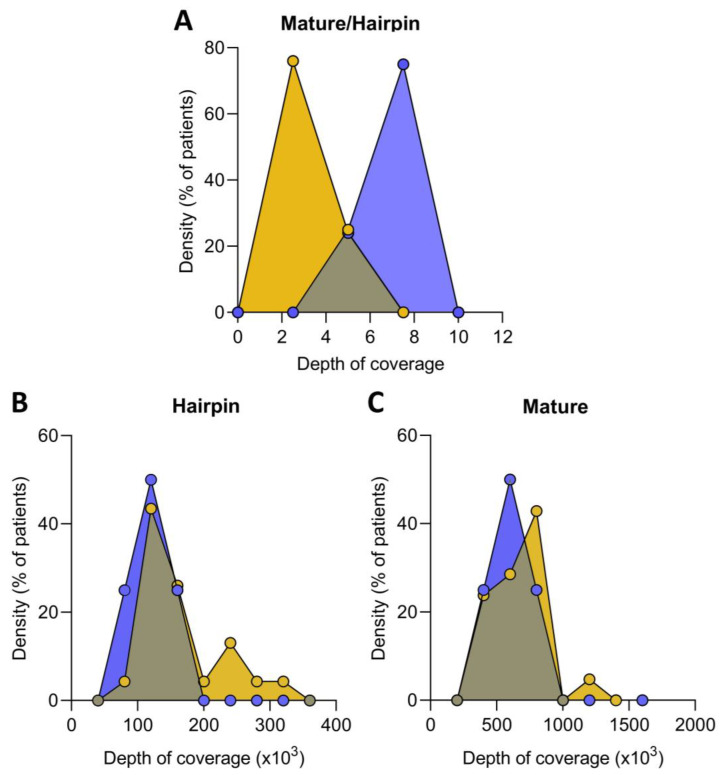
Relative expression of mature and immature microRNA in ischemic cardiomyopathy (ICM, *n* = 22) and controls (*n* = 8) expressed as the ratio of mature/immature microRNA (**A**). Distribution of hairpin (**B**) and mature (**C**) microRNA coverage depth in ICM patients and control. Data are expressed as the fraction of reads mapped against mature microRNA versus hairpin microRNA precursors. ICM patients (orange), control subjects (blue).

Non-coding RNA sequencing was performed to calculate the fraction of reads mapped against mature versus hairpin microRNAs to evaluate the ratio of mature and immature microRNA precursors present in the ICM patients compared to healthy controls. The mature/immature microRNA ratio in ICM patients was lower than in the control group (4.30 ± 0.94 au vs. 5.34 ± 1.07 au, *p* ˂ 0.05), indicating that these patients had a higher presence of immature microRNAs (Figure 1A). Indeed, we found an increase in hairpin microRNAs in ICM versus control (137,091 ± 60,204 vs. 93,887 ± 21,455 counts, *p* ˂ 0.05) (Figure 1B), while mature microRNAs were similar in both groups (607,612 ± 325,107 vs. 518,684 ± 202,203 counts, *p* ˃ 0.05) (Figure 1C).

Subsequently, an mRNA-seq study was performed to analyze differences in transcriptome between microRNA biogenesis-related genes from ICM and control. We focused on the differential gene expression analysis of the main microRNA mechanisms of transcription, processing, export and maturation. All genes analyzed are described in Table 2. This analysis showed that the lower amount of mature microRNA described is accompanied by a deregulation of the microRNA biogenesis process.

**Figure 2:**
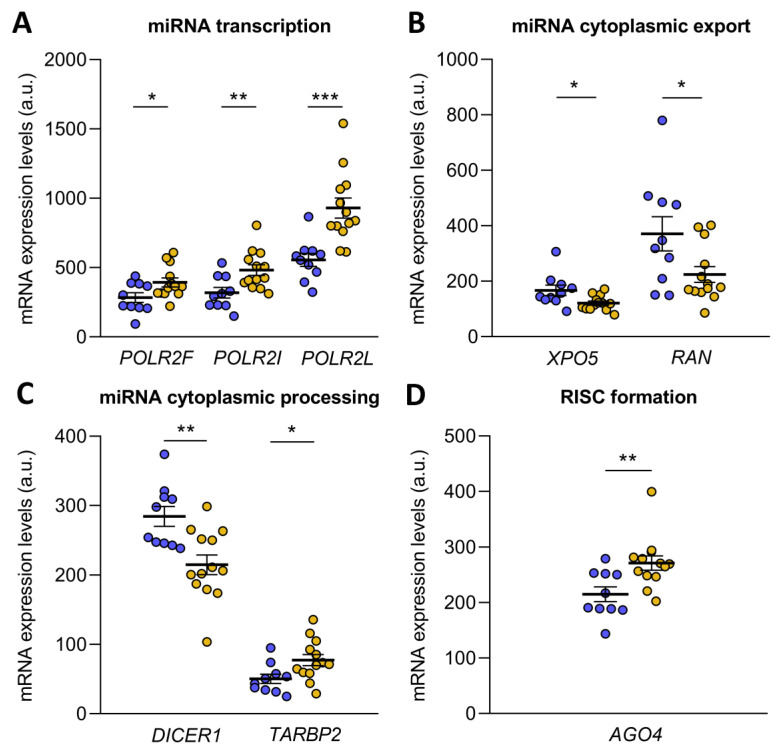
Transcriptome differences in microRNA biogenesis-related genes from ischemic cardiomyopathy patients (ICM, *n* = 13) and control subjects (*n* = 10). Differential gene expression analysis of microRNA transcription (**A**), cytoplasmic export (**B**), cytoplasmic processing (**C**) and RNA-induced silencing complex (RISC) formation (**D**). Data are presented as the mean ± SEM. au, arbitrary units. ICM patients (orange), control subjects (blue). * *p* < 0.05, ** *p* < 0.01, *** *p* < 0.001.

microRNA biogenesis starts with the transcription of pri-microRNA by RNA polymerase II, an enzyme that has 12 subunits, of which *POLR2F* (FC = 1.38, *p* ˂ 0.05), *POLR2I* (FC = 1.51, *p* ˂ 0.01) and *POLR2L* (FC = 1.67, *p* ˂ 0.001) were overexpressed in ICM patients (Figure 2A). Then, the pri-microRNA was cleaved to form the pre-microRNA, which was exported to the cytoplasm in an Exportin-5/RanGTP-dependent manner. Both *XPO5* (FC = −1.38) and *RAN* (FC = −1.66) were underexpressed in ICM patients (*p* ˂ 0.05) (Figure 2B). Upon export to the cytoplasm, pre-microRNA was cleaved by the Endoribonuclease Dicer and TRBP, liberating a small RNA duplex. Both molecules involved in cytoplasmic pre-microRNA processing, *DICER1* (FC = −1.32, *p* ˂ 0.01) and *TARBP2* (a gene that encodes TRBP; FC = 1.54, *p* ˂ 0.05), were found to be deregulated in ischemic origin heart failure observed in the patients (Figure 2C). The small RNA duplex generated by the Endoribonuclease Dicer was subsequently loaded onto an AGO protein to form the RNA-induced silencing complex (RISC), the effector complex of microRNA-induced gene silencing. Of all the Argonaute proteins, only *AGO4* (FC = 1.26, *p* ˂ 0.01) showed an overexpression in ICM patients (Figure 2D).

### 3.3. Cellular Redox Homeostasis Modulation in ICM Patients: Interplay between Oxidative Stress and microRNA

Emerging evidence suggests that microRNA biogenesis can be affected by oxidative stress, and so, an exhaustive literature review was carried out in order to identify the set of microRNAs described as redoximiRs, microRNAs that participate in redox responses both as direct regulatory molecules of the post-transcriptional expression of several pathways and as indirect modulators of the redox homeostatic response, and those microRNAs whose biogenesis is directly modulated by reactive oxygen species (Table S1). The microRNA expression data obtained in the ncRNA-seq were used to compare the microRNA profile of ICM patients to the control group.

**Figure 3:**
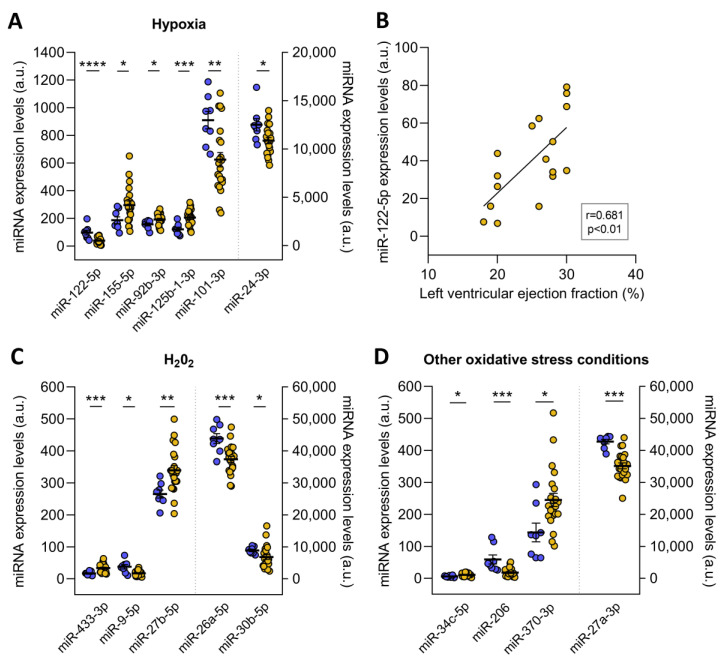
RedoximiRs regulated by stress conditions and differentially expressed in patients with ischemic cardiomyopathy (ICM, *n* = 22) compared to control subjects (*n* = 8). RedoximiRs regulated by hypoxia (**A**). Relationship of miR-122-5p with left ventricular ejection fraction (**B**). RedoximiRs regulated by the presence of H_2_O_2_ (**C**) and redoximiRs regulated by other oxidative stress conditions (**D**). Data are presented as the mean ± SEM. au, arbitrary units. ICM patients (orange), control (blue). * *p* < 0.05, ** *p* < 0.01, *** *p* < 0.001, **** *p* < 0.0001.

The redoximiRs that we identified as differentially expressed are involved in the regulation of ROS production, apoptosis, or DNA repair and are regulated by stress conditions such as hypoxia and H_2_O_2_ (Figure 3). On the one hand, we found altered microRNA in ICM patients that are regulated under conditions of hypoxia: miR-122-5p (FC = −2.41, *p* ˂ 0.001), miR-155-5p (FC = 1.59, *p* ˂ 0.05), miR-92b-3p (FC = 1.23, *p* ˂ 0.05), miR-125b-1-3p (FC = 1.69, *p* ˂ 0.001), miR-101-3p (FC = −1.46, *p* ˂ 0.01), and miR-24-3p (FC = −1.15, *p* ˂ 0.05) (Figure 3A). Interestingly, we observed significant correlations between miR-122-5p and left ventricular ejection fraction (r = 0.681, *p* ˂ 0.01) (Figure 3B). On the other hand, these patients also showed deregulated expression of the miR-433-3p (FC = 1.99, *p* ˂ 0.001), miR-9-5p (FC = −2.14, *p* ˂ 0.05), miR-27b-5p (FC = 1.28, *p* ˂ 0.01), miR-26a-5p (FC = −1.17, *p* ˂ 0.001), and miR-30b-5p (FC = −1.30, *p* ˂ 0.05), which are microRNAs that are H_2_O_2_-sensitive (Figure 3C). Finally, we found changes in miR-34c-5p (FC = 1.83, *p* ˂ 0.05), miR-206 (FC = −3.27, *p* ˂ 0.001), miR-370-3p (FC = 1.71, *p* ˂ 0.05), and miR-27a-3p (FC = −1.22, *p* ˂ 0.001) in ICM patients, which are redoximiRs regulated by other oxidative conditions (Figure 3D).

### 3.4. Construction of the microRNA−mRNA Regulatory Network

**Figure 4:**
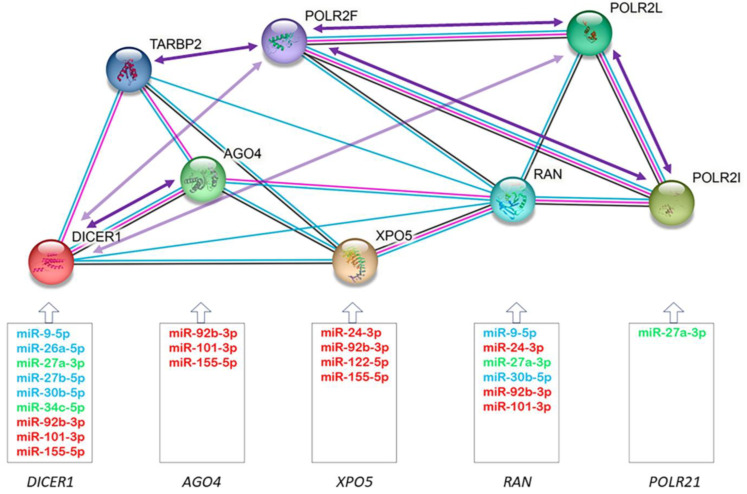
Schematic of the regulatory network showing the validated targets of redoximiRs (miRTarBAse and TaRbase) among differentially expressed genes, protein–protein interactions (STRING), and correlations in the expression of the genes of the microRNA biogenesis pathway. Experimentally validated redoximiRs targets obtained from the miRTarBase and TaRbase databases are shown: redoximiRs regulated under conditions of hypoxia (red), H_2_O_2_ (light blue) and other conditions (light green). Edges represent protein–protein associations: known interactions from curated databases (light blue) and experimentally determined (pink) and co-expression (black). Arrows represent positive (dark purple) or negative (light purple) correlations between mRNA expressions.

Through the miRTarBase and TaRbase databases, we established a microRNA-mRNA regulatory network, where we observed that 73% of redoximiRs altered in the ICM patients regulated one or more of the differentially expressed genes of the microRNA biogenesis pathway (Figure 4). This figure also shows a protein–protein interaction network’s functional enrichment analysis using the String tool, the correlations in the expression of the redoximiRs (Table 3) and the genes of the microRNA biogenesis pathway (Table 4). It should be noted that the export of microRNAs to the cytoplasm (*XPO5* and *RAN*) and their cytoplasmic processing (*DICER1*) were regulated by more than 60% of redoximiRs in both cases, while only 20% of redoximiRs regulated the formation of RISC (*AGO4*) and 1% regulated the transcription of pri-microRNA by RNA polymerase II (*POLR2I*).

### 3.5. Effect of Hypoxia on microRNA Biogenesis: Export to the Cytoplasm and Cytoplasmic Processing

**Figure 5:**
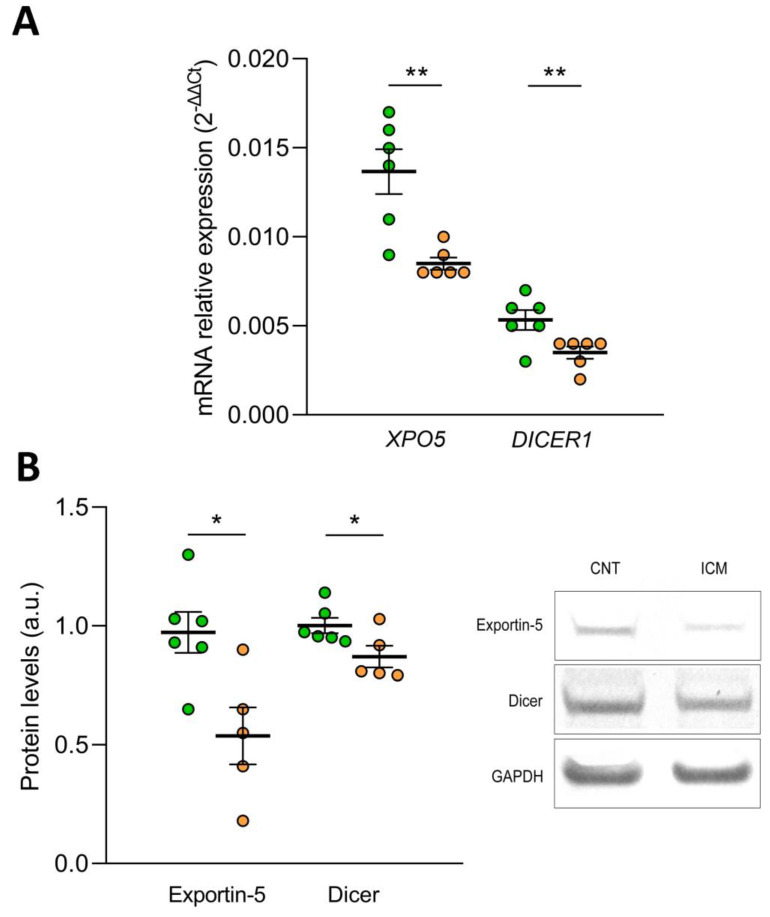
Effect of hypoxia on the expression of Exportin-5/XPO5 and Endoribonuclease Dicer/DICER1 in human cardiomyocyte cell line AC16 at mRNA by RT-qPCR (**A**) and protein level by Western Blot (**B**). Data are presented as the mean ± SEM. au, arbitrary units. AC16 under hypoxic conditions (*n* = 6, orange). AC16 under normoxic conditions (*n* = 6, green). * *p* < 0.05, ** *p* < 0.01.

To elucidate the effect of oxidative stress on the biogenesis of microRNAs, we determined the mRNA and protein expression levels of Exportin-5 and the Endoribonuclease Dicer in the AC16 cell line under hypoxic conditions. We found that after 48 h of hypoxia, the mRNA levels of *XPO5* (FC= −1.65) and *DICER1* (FC = −1.55) were reduced in the human cardiomyocyte cell line AC16 (*p* ˂ 0.01) (Figure 5A). In addition, the protein levels of both molecules were also affected by hypoxia, both Exportin-5 (FC = −1.81) and Endoribonuclease Dicer (FC = −1.15) were downregulated (*p* ˂ 0.05) (Figure 5B).

## 4. Discussion

Numerous studies described abnormal expression levels of microRNA in ICM that involved differential expression of their target genes, implicating them in the pathophysiology of this syndrome [9,10,35]. However, little is known about the underlying causes of microRNA disruption in ICM and whether microRNA maturation is compromised. For this purpose, we investigated the state of microRNA maturity and the expression of the major microRNA biogenesis pathway components. This study demonstrated that global microRNA maturation is dysregulated in human ICM and shed new light on the causes and potential implications of redoximiRs dysregulation. We provided evidence that both changes in the microRNA biogenesis pathway and in redoximiRs, particularly the regulation by hypoxia, could participate in microRNA maturation defects.

To our knowledge, this is the first study that globally analyzed the state of microRNA maturity in ICM or in any pathology in general, while studies generally focused on the analysis of a handful of microRNAs [13]. Our analysis showed a global lower amount of mature microRNA in ICM patients, consistent with previous, mainly cancer, publications based on microRNA clusters [13]. Several authors proposed that in cancer mutation or aberrant expression of any component of the microRNA biogenesis machinery could lead to abnormal microRNA expression [36]. However, although some specific maturation molecules were independently studied, the whole biogenesis pathway was not previously studied. Our cardiac analysis showed that the overall lower amount of mature microRNA we described in ICM was accompanied by a deregulation of the microRNA biogenesis process at four levels: transcription of pri-microRNA by RNA polymerase II, export of pre-microRNA to the cytoplasm, processing of cytoplasmic pre-microRNA and formation of the RISC complex. Additionally, the expression levels of several microRNAs can also be influenced by oxidative stress, an important contributing factor to many diseases including ICM. In turn, microRNAs may control the expression of redox sensors, change important components of the cellular antioxidants and influence DNA repair mechanism [15]. In patients with ICM, we noticed that many of these microRNAs, known as redoximiRs, presented an alteration in their expression levels, mainly regulated by hypoxia, and that most of them targeted one or more of the differentially expressed genes of the microRNA biogenesis pathway.

The transcription of microRNA genes was carried out by RNA polymerase II, a 12-subunit enzyme essential in the transcription process. Increased expression of its subunits may reflect a higher transcriptional activity, and although there are few studies associating its altered expression with disease pathophysiology, beyond elucidating the understanding of the transcription process, it appeared to be disease-specific [37,38]. As far as we know, the relationship between polymerase alteration and ICM was not previously reported. The overexpression of *POLR2F*, *POLR2I* and *POLR2I* that we observed in ICM also showed a good relationship between the three subunits, which could be a response to a lower overall maturation of microRNAs. This premise was also supported by the relationship shown by the expression of these subunits with the expression of the cytoplasmic pre-microRNA processing molecules *DICER1* and *TARBP2* in ICM patients. *DICER1* plays an important role in the maintenance of the cellular response to hypoxia, significantly reducing its protein levels under hypoxic conditions [17], as we observed in the AC16 cell line. Interestingly, *DICER1* knockdown, which was previously associated with the development of heart failure [39], is critical for canonical microRNA biogenesis [13]. Therefore, we observed an underexpression in ICM patients with marked reduction in microRNA maturation. Nevertheless, although we observed a dysregulation of *TARBP2*, it appeared to have no significant effect on overall microRNA abundance but affected Dicer processing in a subset of pre-microRNAs [40].

Regardless of whether there may be an increase or decrease in microRNA transcription, its transport from the nucleus to the cytoplasm is necessary to continue cytoplasmic processing to produce the mature microRNAs, considering it a rate-limiting step during microRNA biogenesis. Any alteration of *XPO5* can impair its nucleus-to-cytoplasmic transport ability, leading to downregulation of mature microRNAs [13,41]. We showed a reduction in both *XPO5* and *RAN* in ICM patients, and confirmed that oxidative stress also regulates microRNA export, observing in AC16 a decrease in both *XPO5* and Exportin-5 levels under hypoxic conditions. AC16 are stable proliferating cell lines, which exhibit ultrastructural, molecular genetics and immunocytochemical characteristics of human cardiomyocytes. They are widely used as an in vitro model to study the function and expression of human cardiac genes, during normal and pathological conditions at the cellular, organellar and molecular levels [42].

The redoximiRs that we found to be differentially expressed are controlled by stress conditions, namely hypoxia, and are involved in the regulation of ROS generation, apoptosis, or DNA repair. Furthermore, several validated targets of these differentially expressed microRNAs correspond to genes from the microRNA biogenesis pathway. Thus, the links between ICM hypoxia and microRNA expression raise the possibility of a general effect of hypoxia on microRNA biogenesis and function. In this sense, altered microRNA biogenesis could modify the availability and functionality of microRNA becoming a causal factor of the adverse effects of hypoxia in ICM patients. Accordingly, we observed a strong relationship between miR-122-5p expression, a microRNA regulated by hypoxia and ventricular dysfunction in ICM patients.

The wide variation in subjects and their treatments, some of which may affect the results, is a major limitation of research that focuses on cardiac tissues from end-stage human heart failure. Additionally, the samples analyzed were mainly from men, due to the higher prevalence of ICM. Consequently, our research population had a homogeneous etiology, and all of the patients examined in this study were receiving medical treatment in accordance with the recommendations of the European Society of Cardiology [20]. In addition, it is essential to underline the significance of conducting this investigation on a sizable number of ICM samples from explanted human hearts undergoing cardiac transplantation and control donors. AC16 Human Cardiomyocyte Cell Line is a proliferating cell line that was derived from the fusion of primary cells from adult human ventricular heart tissues with SV40 transformed, uridine auxotroph human fibroblasts, devoid of mitochondrial DNA. Although in vitro data need to be interpreted with caution, these analyses provide valuable information as a first step to provide concrete answers before further in vivo studies can be performed.

## 5. Conclusions

We showed that microRNA biogenesis is strongly compromised in the human ICM. We evidenced a decrease in microRNA maturation mainly linked to a decrease in *XPO5* mediated pre-microRNA export and a lower *DICER1*-mediated processing, together with a general effect of hypoxia through deregulation of the biogenesis pathway and redoximiRs. Consequently, the availability and functionality of microRNA could become a causal factor of the adverse effects of hypoxia in ICM patients, highlighting the role of miR-122-5p. Since most human protein-coding genes are likely under microRNA control, manipulation of microRNA activity may influence the course of a disease, improving health-related outcomes.

## Figures and Tables

**Table 1:** Clinical characteristics of ischemic cardiomyopathy patients.

	ncRNA-seq	mRNA-seq
	ICM (*n* = 22)	ICM (*n* = 13)
Age (years)	55 ± 8	54 ± 8
Gender male (%)	100	100
*NYHA* class	III–IV	III–IV
BMI (kg/m^2^)	26 ± 3	27 ± 4
Haemoglobin (mg/dL)	14 ± 2	14 ± 3
Haematocrit (%)	41 ± 6	41 ± 6
Total cholesterol (mg/dL)	174 ± 45	162 ± 41
Prior hypertension (%)	40	33
Prior smoking (%)	81	92
Diabetes mellitus (%)	45	42
LVEF (%)	24 ± 5	25 ± 5
LVESD (mm)	53 ± 8	57 ± 8
LVEDD (mm)	62 ± 9	65 ± 8

ICM, ischemic cardiomyopathy; *NYHA*, New York Heart Association; BMI, body mass index; LVEF, ejection fraction; LVESD, left ventricular end-systolic diameter; LVEDD, left ventricular end-diastolic diameter.

**Table 2:** Genes involved in the microRNA biogenesis pathway.

Localization	Function	Gene	Protein
**NUCLEUS**	Transcription	*POLR2A* *POLR2B* *POLR2C* *POLR2D* *POLR2E* ***POLR2F*** *POLR2G* *POLR2H* ***POLR2I*** *POLR2J* *POLR2K* ***POLR2L***	RNA Pol II RPB1 subunit RNA Pol II RPB2 subunit RNA Pol II RPB3 subunit RNA Pol II RPB4 subunit RNA Pol II RPABC1 subunit RNA Pol II RPABC2 subunit RNA Pol II RPB7 subunit RNA Pol II RPABC3 subunit RNA Pol II RPB9 subunit RNA Pol II RPB11-a subunit RNA Pol II RPABC4 subunit RNA Pol II RPABC5 subunit
	Nuclear pri-microRNA processing	*DGCR8* *DROSHA*	Microprocessor complex subunit DGCR8 Ribonuclease 3
**NUCLEAR MEMBRANE**	Nucleo-cytoplasmic export	***XPO5*** ***RAN***	Exportin 5 RAN GTPase
**CYTOPLASM**	Cytoplasmic pre-microRNA processing	***DICER1*** ***TARBP2***	Endoribonuclease Dicer RISC-loading complex subunit TARBP2
	RNA-induced silencing complex formation	*AGO1* *AGO2* *AGO3* ***AGO4***	Argonaute protein 1 Argonaute protein 2 Argonaute protein 3 Argonaute protein 4

Genes highlighted in bold showed altered expression in patients with ischemic cardiomyopathy compared to the control group.

**Table 3:** Relationships between altered redoximiRs in patients with ischemic cardiomyopathy.

			microRNA Regulated by	r	*p*
**microRNA regulated by hypoxia**	miR-122-5p	miR-92-3p miR-125b-1-3p miR-433-3p miR-30b-5p miR-370-3p	Hypoxia Hypoxia H_2_O_2_ H_2_O_2_ Other	0.558 0.545 0.550 −0.477 0.548	˂0.05 ˂0.05 ˂0.05 ˂0.05 ˂0.05
	miR-92-3p	miR-125b-1-3p miR-101-3p miR-433-3p	Hypoxia Hypoxia H_2_O_2_	0.548 −0.454 0.655	˂0.01 ˂0.05 ˂0.001
	miR-125b-1-3p	miR-101-3p miR-433-3p miR-27b-5p miR-30b-5p miR-206	Hypoxia H_2_O_2_ H_2_O_2_ H_2_O_2_ Other	−0.487 0.447 0.456 −0.767 −0.449	˂0.05 ˂0.05 ˂0.05 ˂0.001 ˂0.05
	miR-101-3p	miR-433-3p miR-9-5p miR-27b-5p miR-30b-5p miR-27a-3p	H_2_O_2_ H_2_O_2_ H_2_O_2_ H_2_O_2_ Other	−0.566 0.565 −0.503 0.704 0.629	˂0.01 ˂0.01 ˂0.05 ˂0.001 ˂0.01
**microRNA regulated by H_2_O_2_**	miR-433-3p	miR-30b-5p miR-370-3p	H_2_O_2_ Other	−0.460 0.576	˂0.05 ˂0.01
	miR-27b-5p	miR-34c-5p	Other	−0.446	˂0.05
	miR30b-5p	miR-27b-5p	H_2_O_2_	−0.573	˂0.01

**Table 4:** Relationships between differentially expressed genes of the microRNA biogenesis pathway in ischemic cardiomyopathy.

			Function	r	*p*
**Transcription**	*POLR2F*	*POLR2I* *POLR2L* *DICER1* *TARBP2*	Transcription Transcription Cytoplasmic processing Cytoplasmic processing	0.595 0.684 −0.564 0.769	˂0.05 ˂0.01 ˂0.05 ˂0.01
	*POLR2L*	*POLR2I* *DICER1*	Transcription Cytoplasmic processing	0.738 −0.576	˂0.01 ˂0.05
**Cytoplasmic processing**	*DICER1*	*AGO4*	RISC formation	0.570	˂0.05

## Data Availability

The mRNA-seq data discussed in this publication were deposited in NCBI’s Gene Expression Omnibus [27] and are accessible through GEO Series Accession Number GSE55296 (http://www.ncbi.nlm.nih.gov/geo/query/acc.cgi?acc=GSE55296, accessed on 28 April 2014).

## Supplementary Materials

The following supporting information can be downloaded at: https://www.mdpi.com/article/10.3390/antiox12071337/s1, Table S1: microRNAs regulating antioxidant responses and/or being regulated by ROS (redoximiRs) analyzed. References [16,19,43,44,45,46,47,48,49,50,51,52,53,54,55,56,57,58,59,60,61,62,63,64,65,66,67,68,69,70,71,72,73,74,75,76,77,78,79,80,81,82,83,84,85,86,87,88,89,90] are cited in the supplementary materials.
