# Supplementary material for: Altered MicroRNA Maturation in Ischemic Hearts: Implication of Hypoxia on XPO5 and DICER1 Dysregulation and RedoximiR State
Source: Antioxidants (Basel). 2023 Jun 24;12(7):1337. doi: 10.3390/antiox12071337 (PMC10376795; doi:10.3390/antiox12071337)
Supplement: Supplementary file 1 [file antioxidants-12-01337-s001.zip › antioxidants-2396010-supplementary.pdf]

Altered miRNA Maturation in Ischemic Hearts: Implication of Hypoxia on XPO5 and DICER1 Dysregulation and RedoximiR State

Table S1. miRNAs regulating antioxidant responses and/or being regulated by ROS (redoximiRs) analysed.

**miRNA**	**Molecular target**	**Function**	**Reference**
**miR-101-3p**	Cul3	Hypoxia elevates the expression of miR-101 which inhibited the expression of cul3. A reduction in the level of Cul3 activates the Nrf2/HO-1 axis	[1]
**miR-122-5p**	CDK4-PSMD10 pathway	miR-122 negatively regulates the UPR through the CDK4-PSMD10 pathway	[2,3]
miR-124-3p	CYBB	miR-124-3p negatively regulate CYBB expression and NOX2 protein level. miR-124-5p/NOX2 axis modulates NOX-mediated ROS production	[4]

**miR-125b-1-3p**		miR-125b-1-3p acts as a cardioprotector in ischemia-reperfusion injury	[5,6]
miR-128-3p	MAFG	miR-128 expression is modulated by hypoxic conditions and negatively regulates the expression of MAFG and could affect MAFG-related genes such as HMOX-1 and x-CT	[7]
miR-130a-3p	APE1	Lower miR-130a could generate a more extensive response to oxidative stress, which in turn could elevate Ape1	[8]
miR-132	TGF-β1<br>smad3	miR-132 inhibits oxidative stress and apoptosis and decreases the expression of TGF-β1	[9]

		and smad3	
miR-141	ZEB1 targets miR-141	miR-141 is upregulated by oxidative stress and is inhibited by ZEB1	[10]
miR-144-3p	NFR2	miR-144 reduce nuclear factor-erythroid 2-related factor 2 (NRF2) and is also associated with decreased glutathione regeneration and attenuated antioxidant capacity	[11]
miR-145	SGK1	HIF-1a, under hypoxic conditions, induces overexpression of miR-145. miR-145 protects against hypoxic damage, inducing SGK1 expression	[12]
miR-146a	Catalase	miR-146a mediates catalase suppression	[13]

		in the absence of RIP1	
miR-153	Nfr2	miR-153 is up-regulated in oxidative stress conditions and represses Nrf2	[14]
**miR-155-5p**	Dicer targets miR-155<br>miR-155 targets HK2	miR-155 is regulated by Dicer during chronic hypoxia, and activates HK2 expression and glycolysis	[15]
miR-181a-5p	Bcl-2	miR-181a is induced by H_2O_2 and supresses Bcl-2 expression	[16]
miR-185-5p		Hypoxia induces miR-185 expression. miR-185 inhibits the DNA damage-induced 14-3-3δ signalling pathway	[17]
miR-191-5p	C/EBPβ	miR-191 is induced by hypoxia and represses	[18]

		CCAAT/enhancer binding protein β (C/EBPβ)	
miR-196b-5p	Bach-1	miR-196 down-regulated Bach1 and up-regulated HMOX1 gene expression, and inhibited HCV expression	[19]
miR-199	Hif-1α<br>Sirt1	miR-199 is downregulated during hypoxia and inhibits Hif-1α and Sirt1 expression	[20]
miR-200a-3p	keap1	miR-200a regulates the Keap1/Nrf2 pathway	[21]
miR-200b		miRNA-200b expression is decreases by hypoxia	[22]
miR-200c-3p	ZEB/miR-200 double-negative feedback loop	ZEB factors transcriptionally repress miR-200 family members and these inhibit ZEB	[23]

		expression at the post-transcriptional level. miR-200c is upregulated by oxidative stress	
**miR-206**	SOD1	miR-206 inhibits SOD1 expression, promoting ROS production	[24]
miR-21-5p	NF-κB targets miR-21 miR-21 targets PDCD4	NF-κB positively regulated miR-21 expression under oxidative stress, and PDCD4 was a direct target for miR-21	[25]
miR-210-3p	Several pathways related to the low cellular oxygen	miR-210-3p was highly up-regulated in the nuclear compartment after hypoxic stimulus	[26]
miR-212-3p	SOD2	miR-212-3p inhibits SOD2 expression, promoting ROS production	[27]
miR-23b-5p	POX	miR-23b-5p inhibits	[28]

		POX expression. POX induces apoptosis through the generation of ROS and decreasing HIF signalling	
**miR-24-3p**	HMOX-1<br>Cellular survival pathways (e.g., AKT and ERK)	miR-24 is a hypoxia-sensitive miRNA, represses HMOX-1 expression and induces apoptosis and oxidative stress	[29]
miR-25-3p	MITF<br>SCF<br>bFGF	Oxidative stress could induce the overexpression of miR-25 that reduced the expression of MITF, SCF and bFGF	[30]
**miR-26a-5p**	PTEN	miR-26a is inhibited by H_2O_2, protects against H_2O_2-induced injury by targeting PTEN and prevents H_2O_2-induced apoptosis through re-	[31]

		activation of the Akt/mTOR pathway	
**miR-27a-3p**<br>miR-27b-3p	PHB1<br>Nrf2	miR27a and miR27b downregulate Nrf2 and PHB1 expression induced by c-Myc	[32]
**miR-27b-5p**	NF-kB	H_2O_2 inhibits the expression of miR-27b and it supresses NF-kB pathway	[33]
**miR-30b-5p**	Catalase	H_2O_2 upregulated the expression of miR-30b-5p, which inhibited the expression of endogenous catalase	[34]
miR-31-5p	SIRT3	miR-31 targets SIRT3 to repress its expression, being involved in ROS regulation	[35]
miR-34a-5p	Sirt1<br>Mgst1<br>Sp1<br>Nrf2	miR-34a represses expression, of Sirt1 and Mgst1 and its upstream	[36]

		transcription factors Sp1 and Nrf2	
miR-34b<br>**miR-34c-5p**	DJ-1<br>PARKIN	miR-34b and miR-34c reduction results in a decreased expression of DJ1 and PARKIN, and is associated with mitochondrial dysfunction and reactive oxygen species production	[37]
miR-361-3p	caspase-2/-8/-9	Hypoxia decreased miR-361-3p expression, and it overexpression mitigated hypoxia injury by inhibiting apoptosis via targeting apoptosis initiators caspase-2/-8/-9	[38]
**miR-370-3p**	MGMT	miR-370-3p downregulates MGMT gene expression and	[39]

		inhibits DNA repair	
miR-382-5p	PTEN	Hypoxia and HIF-1α induce the expression of miR-382-5p. miR-382-5p inhibits PTEN levels and stimulates the AKT/mTOR signalling pathway	[40]
miR-424-5p	CUL2	Hypoxia increases miR-424 that targets cullin 2 (CUL2) stabilizing HIF-α	[41]
miR-429	HIF1A	miR-429 expression is induced by HIF-1 and by hypoxia. miR-429 inhibits HIF1A and indirectly affects VEGFA mRNA	[42]
**miR-433-3p**	γ-glutamyl-cysteine ligase	miR-433 decreases glutathione levels by targeting glutamate cysteine ligase; increases oxidative stress	[43]
miR-4673	OGG1	miR-4673	[44]

		downregulates OGG1 levels, inducing ROS production	
miR-506-3p	NF-kB p65<br>P53 targets miR-506-3p	miR-506 inhibits NF-kB p65 expression, induces ROS accumulation, and then activates p53. In addition, p53 promotes miR-506 expression level, indicating that miR-506 mediates cross-talk between p53, NF-κB p65 and ROS	[45]
miR-509-5p	SOD2	miR-509-5p inhibits SOD2	[46]
miR-551b-3p	Catalase	miR-551b-3p inhibited the expression of catalase and potentiated ROS accumulation	[47]
miR-765	VEGFA	miR-765 was severely downregulated after	[48]

		hypoxia and decreases the levels of VEGFA, AKT1 and SRC-α	
**miR-9-5p**	NOX4<br>TGFBR2	miR-9-5p is up-regulated in oxidative stress conditions and inhibits TGFBR2 and NOX4	[49]
**miR-92b-3p**	TSC1	miR-92b-3p is upregulated by hypoxia and suppresses TSC1 expression	[50]

miRNAs highlighted in bold have altered expression in patients with ischemic cardiomyopathy compared to the control group. The molecular target and functions described here are focused on those that relate miRNA, its biogenesis and oxidative stress.

1. Kim, J.H.; Lee, K.S.; Lee, D.K.; Kim, J.; Kwak, S.N.; Ha, K.S.; Choe, J.; Won, M.H.; Cho, B.R.; Jeoung, D.; et al. Hypoxia-responsive microRNA-101 promotes angiogenesis via heme oxygenase-1/vascular endothelial growth factor axis by targeting cullin 3. *Antioxid Redox Signal* **2014**, *21*, 2469-2482.
2. Yang, F.; Zhang, L.; Wang, F.; Wang, Y.; Huo, X.S.; Yin, Y.X.; Wang, Y.Q.; Zhang, L.; Sun, S.H. Modulation of the unfolded protein response is the core of microRNA-122-involved sensitivity to chemotherapy in hepatocellular carcinoma. *Neoplasia* **2011**, *13*, 590-600.
3. Zhi, F.; Shao, N.; Xue, L.; Xu, Y.; Kang, X.; Yang, Y.; Xia, Y. Characteristic MicroRNA Expression Induced by delta-Opioid Receptor Activation in the Rat Liver Under Prolonged Hypoxia. *Cell Physiol Biochem* **2017**, *44*, 2296-2309.
4. Berardino, B.G.; Fesser, E.A.; Canepa, E.T. Perinatal protein malnutrition alters expression of miRNA biogenesis genes Xpo5 and Ago2 in mice brain. *Neurosci Lett* **2017**, *647*, 38-44.
5. Varga, Z.V.; Zvara, A.; Farago, N.; Kocsis, G.F.; Pipicz, M.; Gaspar, R.; Bencsik, P.; Gorbe, A.; Csonka, C.; Puskas, L.G.; et al. MicroRNAs associated with ischemia-reperfusion

injury and cardioprotection by ischemic pre- and postconditioning: protectomiRs. *Am J Physiol Heart Circ Physiol* **2014**, *307*, H216-227.
6. Szabo, M.R.; Gaspar, R.; Pipicz, M.; Zsindely, N.; Dioszegi, P.; Sarkozy, M.; Bodai, L.; Csont, T. Hypercholesterolemia Interferes with Induction of miR-125b-1-3p in Preconditioned Hearts. *Int J Mol Sci* **2020**, *21*.
7. Caggiano, R.; Cattaneo, F.; Moltedo, O.; Esposito, G.; Perrino, C.; Trimarco, B.; Ammendola, R.; Faraonio, R. miR-128 Is Implicated in Stress Responses by Targeting MAFG in Skeletal Muscle Cells. *Oxid Med Cell Longev* **2017**, *2017*, 9308310.
8. Chen, H.; Li, X.; Li, W.; Zheng, H. miR-130a can predict response to temozolomide in patients with glioblastoma multiforme, independently of O6-methylguanine-DNA methyltransferase. *J Transl Med* **2015**, *13*, 69.
9. Liu, X.; Tong, Z.; Chen, K.; Hu, X.; Jin, H.; Hou, M. The Role of miRNA-132 against Apoptosis and Oxidative Stress in Heart Failure. *Biomed Res Int* **2018**, *2018*, 3452748.
10. Brabletz, S.; Brabletz, T. The ZEB/miR-200 feedback loop--a motor of cellular plasticity in development and cancer? *EMBO Rep* **2010**, *11*, 670-677.
11. Sangokoya, C.; Telen, M.J.; Chi, J.T. microRNA miR-144 modulates oxidative stress tolerance and associates with anemia severity in sickle cell disease. *Blood* **2010**, *116*, 4338-4348.
12. Sun, N.; Meng, F.; Xue, N.; Pang, G.; Wang, Q.; Ma, H. Inducible miR-145 expression by HIF-1a protects cardiomyocytes against apoptosis via regulating SGK1 in simulated myocardial infarction hypoxic microenvironment. *Cardiol J* **2018**, *25*, 268-278.
13. Wang, Q.; Chen, W.; Bai, L.; Chen, W.; Padilla, M.T.; Lin, A.S.; Shi, S.; Wang, X.; Lin, Y. Receptor-interacting protein 1 increases chemoresistance by maintaining inhibitor of apoptosis protein levels and reducing reactive oxygen species through a microRNA-146a-mediated catalase pathway. *J Biol Chem* **2014**, *289*, 5654-5663.
14. Narasimhan, M.; Riar, A.K.; Rathinam, M.L.; Vedpathak, D.; Henderson, G.; Mahimainathan, L. Hydrogen peroxide responsive miR153 targets Nrf2/ARE cytoprotection in paraquat induced dopaminergic neurotoxicity. *Toxicol Lett* **2014**, *228*, 179-191.
15. Yao, M.; Wang, X.; Tang, Y.; Zhang, W.; Cui, B.; Liu, Q.; Xing, L. Dicer mediating the expression of miR-143 and miR-155 regulates hexokinase II associated cellular response to hypoxia. *Am J Physiol Lung Cell Mol Physiol* **2014**, *307*, L829-837.
16. Liu, G.; Li, Y.; Gao, X.G. microRNA-181a is upregulated in human atherosclerosis plaques and involves in the oxidative stress-induced endothelial cell dysfunction through direct targeting Bcl-2. *Eur Rev Med Pharmacol Sci* **2016**, *20*, 3092-3100.
17. Zhang, D.; Lee, H.; Cao, Y.; Dela Cruz, C.S.; Jin, Y. miR-185 mediates lung epithelial cell death after oxidative stress. *Am J Physiol Lung Cell Mol Physiol* **2016**, *310*, L700-710.
18. Yu, S.; Lu, Y.; Zong, M.; Tan, Q.; Fan, L. Hypoxia-induced miR-191-C/EBPbeta signaling regulates cell proliferation and apoptosis of fibroblast-like synoviocytes from patients with rheumatoid arthritis. *Arthritis Res Ther* **2019**, *21*, 78.
19. Hou, W.; Tian, Q.; Zheng, J.; Bonkovsky, H.L. MicroRNA-196 represses Bach1 protein and hepatitis C virus gene expression in human hepatoma cells expressing hepatitis C viral proteins. *Hepatology* **2010**, *51*, 1494-1504.
20. Rane, S.; He, M.; Sayed, D.; Vashistha, H.; Malhotra, A.; Sadoshima, J.; Vatner, D.E.; Vatner, S.F.; Abdellatif, M. Downregulation of miR-199a derepresses hypoxia-inducible factor-1alpha and Sirtuin 1 and recapitulates hypoxia preconditioning in cardiac myocytes. *Circ Res* **2009**, *104*, 879-886.
21. Eades, G.; Yang, M.; Yao, Y.; Zhang, Y.; Zhou, Q. miR-200a regulates Nrf2 activation by targeting Keap1 mRNA in breast cancer cells. *J Biol Chem* **2011**, *286*, 40725-40733.
22. Riehle, C.; Sieweke, J.T.; Bakshi, S.; Ha, C.M.; Junker Udesen, N.L.; Moller-Helgestad, O.K.; Froese, N.; Berg Ravn, H.; Bahre, H.; Geffers, R.; et al. miRNA-200b-A Potential

Biomarker Identified in a Porcine Model of Cardiogenic Shock and Mechanical Unloading. *Front Cardiovasc Med* **2022**, *9*, 881067.
23. Magenta, A.; Cencioni, C.; Fasanaro, P.; Zaccagnini, G.; Greco, S.; Sarra-Ferraris, G.; Antonini, A.; Martelli, F.; Capogrossi, M.C. miR-200c is upregulated by oxidative stress and induces endothelial cell apoptosis and senescence via ZEB1 inhibition. *Cell Death Differ* **2011**, *18*, 1628-1639.
24. Zhang, Y.; Zheng, S.; Geng, Y.; Xue, J.; Wang, Z.; Xie, X.; Wang, J.; Zhang, S.; Hou, Y. MicroRNA profiling of atrial fibrillation in canines: miR-206 modulates intrinsic cardiac autonomic nerve remodeling by regulating SOD1. *PLoS One* **2015**, *10*, e0122674.
25. Wei, C.; Li, L.; Kim, I.K.; Sun, P.; Gupta, S. NF-kappaB mediated miR-21 regulation in cardiomyocytes apoptosis under oxidative stress. *Free Radic Res* **2014**, *48*, 282-291.
26. Turunen, T.A.; Roberts, T.C.; Laitinen, P.; Vaananen, M.A.; Korhonen, P.; Malm, T.; Yla-Herttuala, S.; Turunen, M.P. Changes in nuclear and cytoplasmic microRNA distribution in response to hypoxic stress. *Sci Rep* **2019**, *9*, 10332.
27. Meng, X.; Wu, J.; Pan, C.; Wang, H.; Ying, X.; Zhou, Y.; Yu, H.; Zuo, Y.; Pan, Z.; Liu, R.Y.; et al. Genetic and epigenetic down-regulation of microRNA-212 promotes colorectal tumor metastasis via dysregulation of MnSOD. *Gastroenterology* **2013**, *145*, 426-436 e421-426.
28. Liu, W.; Zabirnyk, O.; Wang, H.; Shiao, Y.H.; Nickerson, M.L.; Khalil, S.; Anderson, L.M.; Perantoni, A.O.; Phang, J.M. miR-23b targets proline oxidase, a novel tumor suppressor protein in renal cancer. *Oncogene* **2010**, *29*, 4914-4924.
29. Fiedler, J.; Stohr, A.; Gupta, S.K.; Hartmann, D.; Holzmann, A.; Just, A.; Hansen, A.; Hilfiker-Kleiner, D.; Eschenhagen, T.; Thum, T. Functional microRNA library screening identifies the hypoxamir miR-24 as a potent regulator of smooth muscle cell proliferation and vascularization. *Antioxid Redox Signal* **2014**, *21*, 1167-1176.
30. Shi, Q.; Zhang, W.; Guo, S.; Jian, Z.; Li, S.; Li, K.; Ge, R.; Dai, W.; Wang, G.; Gao, T.; et al. Oxidative stress-induced overexpression of miR-25: the mechanism underlying the degeneration of melanocytes in vitiligo. *Cell Death Differ* **2016**, *23*, 496-508.
31. Peng, J.; He, X.; Zhang, L.; Liu, P. MicroRNA-26a protects vascular smooth muscle cells against H2O2-induced injury through activation of the PTEN/AKT/mTOR pathway. *Int J Mol Med* **2018**, *42*, 1367-1378.
32. Yang, H.; Li, T.W.; Zhou, Y.; Peng, H.; Liu, T.; Zandi, E.; Martinez-Chantar, M.L.; Mato, J.M.; Lu, S.C. Activation of a novel c-Myc-miR27-prohibitin 1 circuitry in cholestatic liver injury inhibits glutathione synthesis in mice. *Antioxid Redox Signal* **2015**, *22*, 259-274.
33. Thulasingam, S.; Massilamany, C.; Gangaplara, A.; Dai, H.; Yarbaeva, S.; Subramaniam, S.; Riethoven, J.J.; Eudy, J.; Lou, M.; Reddy, J. miR-27b*, an oxidative stress-responsive microRNA modulates nuclear factor-kB pathway in RAW 264.7 cells. *Mol Cell Biochem* **2011**, *352*, 181-188.
34. Haque, R.; Chun, E.; Howell, J.C.; Sengupta, T.; Chen, D.; Kim, H. MicroRNA-30b-mediated regulation of catalase expression in human ARPE-19 cells. *PLoS One* **2012**, *7*, e42542.
35. Kao, Y.Y.; Chou, C.H.; Yeh, L.Y.; Chen, Y.F.; Chang, K.W.; Liu, C.J.; Fan Chiang, C.Y.; Lin, S.C. MicroRNA miR-31 targets SIRT3 to disrupt mitochondrial activity and increase oxidative stress in oral carcinoma. *Cancer Lett* **2019**, *456*, 40-48.
36. Li, N.; Muthusamy, S.; Liang, R.; Sarojini, H.; Wang, E. Increased expression of miR-34a and miR-93 in rat liver during aging, and their impact on the expression of Mgst1 and Sirt1. *Mech Ageing Dev* **2011**, *132*, 75-85.
37. Minones-Moyano, E.; Porta, S.; Escaramis, G.; Rabionet, R.; Iraola, S.; Kagerbauer, B.; Espinosa-Parrilla, Y.; Ferrer, I.; Estivill, X.; Marti, E. MicroRNA profiling of Parkinson's disease brains identifies early downregulation of miR-34b/c which modulate mitochondrial function. *Hum Mol Genet* **2011**, *20*, 3067-3078.

38. Zhang, J.; Zhou, X.; Sun, J.; Li, M.; Ma, J.; Ge, L. miR-361-3p mitigates hypoxia-induced cardiomyocyte injury via targeting apoptosis initiators caspase-2/-8/-9. *In Vitro Cell Dev Biol Anim* **2022**, *58*, 116-123.
39. Gao, Y.T.; Chen, X.B.; Liu, H.L. Up-regulation of miR-370-3p restores glioblastoma multiforme sensitivity to temozolomide by influencing MGMT expression. *Sci Rep* **2016**, *6*, 32972.
40. Seok, J.K.; Lee, S.H.; Kim, M.J.; Lee, Y.M. MicroRNA-382 induced by HIF-1alpha is an angiogenic miR targeting the tumor suppressor phosphatase and tensin homolog. *Nucleic Acids Res* **2014**, *42*, 8062-8072.
41. Ghosh, G.; Subramanian, I.V.; Adhikari, N.; Zhang, X.; Joshi, H.P.; Basi, D.; Chandrashekhar, Y.S.; Hall, J.L.; Roy, S.; Zeng, Y.; et al. Hypoxia-induced microRNA-424 expression in human endothelial cells regulates HIF-alpha isoforms and promotes angiogenesis. *J Clin Invest* **2010**, *120*, 4141-4154.
42. Bartoszewska, S.; Kochan, K.; Piotrowski, A.; Kamysz, W.; Ochocka, R.J.; Collawn, J.F.; Bartoszewski, R. The hypoxia-inducible miR-429 regulates hypoxia-inducible factor-1alpha expression in human endothelial cells through a negative feedback loop. *FASEB J* **2015**, *29*, 1467-1479.
43. Espinosa-Diez, C.; Fierro-Fernandez, M.; Sanchez-Gomez, F.; Rodriguez-Pascual, F.; Alique, M.; Ruiz-Ortega, M.; Beraza, N.; Martinez-Chantar, M.L.; Fernandez-Hernando, C.; Lamas, S. Targeting of Gamma-Glutamyl-Cysteine Ligase by miR-433 Reduces Glutathione Biosynthesis and Promotes TGF-beta-Dependent Fibrogenesis. *Antioxid Redox Signal* **2015**, *23*, 1092-1105.
44. Huang, H.L.; Shi, Y.P.; He, H.J.; Wang, Y.H.; Chen, T.; Yang, L.W.; Yang, T.; Chen, J.; Cao, J.; Yao, W.M.; et al. MiR-4673 Modulates Paclitaxel-Induced Oxidative Stress and Loss of Mitochondrial Membrane Potential by Targeting 8-Oxoguanine-DNA Glycosylase-1. *Cell Physiol Biochem* **2017**, *42*, 889-900.
45. Yin, M.; Ren, X.; Zhang, X.; Luo, Y.; Wang, G.; Huang, K.; Feng, S.; Bao, X.; Huang, K.; He, X.; et al. Selective killing of lung cancer cells by miRNA-506 molecule through inhibiting NF-kappaB p65 to evoke reactive oxygen species generation and p53 activation. *Oncogene* **2015**, *34*, 691-703.
46. Song, Y.H.; Wang, J.; Nie, G.; Chen, Y.J.; Li, X.; Jiang, X.; Cao, W.H. MicroRNA-509-5p functions as an anti-oncogene in breast cancer via targeting SOD2. *Eur Rev Med Pharmacol Sci* **2017**, *21*, 3617-3625.
47. Xu, X.; Wells, A.; Padilla, M.T.; Kato, K.; Kim, K.C.; Lin, Y. A signaling pathway consisting of miR-551b, catalase and MUC1 contributes to acquired apoptosis resistance and chemoresistance. *Carcinogenesis* **2014**, *35*, 2457-2466.
48. Salinas-Vera, Y.M.; Gallardo-Rincon, D.; Garcia-Vazquez, R.; Hernandez-de la Cruz, O.N.; Marchat, L.A.; Gonzalez-Barrios, J.A.; Ruiz-Garcia, E.; Vazquez-Calzada, C.; Contreras-Sanzon, E.; Resendiz-Hernandez, M.; et al. Corrigendum: HypoxamiRs Profiling Identify miR-765 as a Regulator of the Early Stages of Vasculogenic Mimicry in SKOV3 Ovarian Cancer Cells. *Front Oncol* **2020**, *10*, 889.
49. Fierro-Fernandez, M.; Busnadiego, O.; Sandoval, P.; Espinosa-Diez, C.; Blanco-Ruiz, E.; Rodriguez, M.; Pian, H.; Ramos, R.; Lopez-Cabrera, M.; Garcia-Bermejo, M.L.; et al. miR-9-5p suppresses pro-fibrogenic transformation of fibroblasts and prevents organ fibrosis by targeting NOX4 and TGFBR2. *EMBO Rep* **2015**, *16*, 1358-1377.
50. Lee, J.; Heo, J.; Kang, H. miR-92b-3p-TSC1 axis is critical for mTOR signaling-mediated vascular smooth muscle cell proliferation induced by hypoxia. *Cell Death Differ* **2019**, *26*, 1782-1795.
